# Effect of proton pump inhibitors on mortality of cirrhotic patients with pneumonia

**DOI:** 10.1371/journal.pone.0216041

**Published:** 2019-04-25

**Authors:** Tsung-Hsing Hung, Chih -Wei Tseng, Chih-Chun Tsai, Hsing-Feng Lee

**Affiliations:** 1 Department of Medicine, Dalin Tzu Chi Hospital, Buddhist Tzu Chi Medical Foundation, Chiayi, Taiwan; 2 School of Medicine, Tzu Chi University, Hualien, Taiwan; 3 Department of Mathematics, Tamkang University, Tamsui, Taiwan; University of Mississippi Medical Center, UNITED STATES

## Abstract

**Objective:**

Pneumonia is life-threatening in patients with liver cirrhosis. Proton pump inhibitors (PPIs) may increase the risk of these patients developing pneumonia. However, whether PPIs increase mortality in patients with cirrhosis and pneumonia remain unknown.

**Methods:**

We used the Taiwan National Health Insurance Database to enroll 1,201 cirrhotic patients with pneumonia without active gastrointestinal bleeding who were receiving PPIs and were hospitalized between January 1, 2010 and December 31, 2013. A one-to-three propensity score match was performed to select a comparison group based on age, gender, and comorbid disorders.

**Results:**

The overall 30-day and 90-day all-cause mortality rates were 13.7% and 26.9% in the PPI group, and 14.3% and 25.1% in the non-PPI group, respectively. After Cox regression model adjusting for age, gender, and comorbid disorders, the hazard ratios of the effect of PPIs on 30-day and 30 to 90-day mortality were 0.94 (95% Confidence Interval [CI], 0.79–1.12, P = 0.468) and 1.26 (95% CI, 1.05–1.52; P = 0.013), respectively.

**Conclusions:**

PPIs were not associated with 30-day mortality among cirrhotic patients with pneumonia but not active gastrointestinal bleeding. However, prolonged PPI therapy may be associated with higher mortality.

## Introduction

Patients with liver cirrhosis are prone to bacterial infections due to their impaired immune status, the increased use of invasive procedures, and alterations in the enteric flora [1, 2]. Bacterial infection is the main cause for hospitalization among patients with liver cirrhosis [3] and contributes to their fourfold greater likelihood of death over patients without cirrhosis [4]. In addition, bacterial infections can trigger and aggravate cirrhosis-related complications, such as hepatic encephalopathy, ascites, variceal bleeding, or hepatic renal syndrome [1–4].

Proton pump inhibitors (PPIs), a potent gastric acid suppressant, are used for gastric acid-related diseases such as gastroesophageal reflux disease and peptic ulcers [5]. In cirrhotic patients, PPIs can also decrease the ulcer related to endoscopic ligation of esophageal varices [6]. PPIs therapy is common in patients with liver cirrhosis [7, 8]. However, current evidence has shown that PPIs can enhance small intestinal bacterial overgrowth (SIBO), leading to bacterial translocation [9, 10]. PPIs can also markedly reduce gastric acid secretion. Gastric acid plays an important role in inhibiting bacterial overgrowth in the stomach. With gastric acid suppression, infectious disease such as pneumonia may occur more easily. Several studies have reported a higher risk of pneumonia after the initiation of PPI therapy [11, 12].

However, the result of using PPIs in cirrhotic patients who have already have pneumonia may be different. In cirrhotic patients with pneumonia, it seems inevitable in clinical practice to use antibiotics to avoid sepsis. Pneumonia is a major risk for cirrhotic patients, having been reported in about 21.4% of cirrhotic patients, and can lead to mortality rates as high as 32–41% [4, 13–15]. It is clinically useful to know the association of PPIs with mortality in hospitalized cirrhotic patients receiving antibiotics. The disadvantage of reducing gastric acid secretions by PPIs may not be a problem according to the antibiotics used. We sought to test the association of PPIs with mortality in cirrhotic patients hospitalized with pneumonia who were also taking antibiotics. Using data from the Taiwan National Health Insurance Research Database, we enrolled a large population of cirrhotic patients with pneumonia to assess the association of oral PPIs on mortality among these patients.

## Materials and methods

### Database and ethical statement

In Taiwan, the Bureau of National Health Insurance (BNHI) administers the National Health Insurance program, which currently covers more than 98% of the Taiwan population. For medical payment, all contracted medical institutions must provide medical records to the BNHI. The BNHI and the National Health Research Institute (NHRI) have used these medical records to establish the National Health Insurance Research Database (NHIRD) for medical research. The dataset we used in the present study was from the NHIRD released from BNHI and NHRI. The application and agreement number in NHRI was 104359. The dataset contained all International Classification of Diseases, 9th Revision, Clinical Modification (ICD-9-CM) codes of hospitalized patients in Taiwan. This study was approved by the Institutional Review Board of the Buddhist Dalin Tzu Chi Hospital (IRB B10403026). The IRB waived the requirement for written informed consent from all patients because the secondary de-identified dataset did not include any personal information.

### Study sample

First, we searched the dataset for patients discharged with a main or accessory diagnosis of cirrhosis (ICD-9-CM codes 571.5 or 571.2) between January 1, 2010 and December 31, 2013. Of these patients, we enrolled those who also had a diagnosis of pneumonia (ICD-9-CM codes 481–487) [16]. If the patients had multiple hospitalizations for respiratory infections during this period, only the first episode was included in the analysis. Second, we excluded patients with active gastrointestinal bleeding during hospitalization. This included patients with upper gastrointestinal tract bleeding (UGIB) (ICD-9-CM codes 531.0, 531.2, 531.4, 531.6, 532.0, 532.2, 532.4, 532.6, 533.0, 533.2, 533.4, and 533.6), esophageal variceal bleeding (ICD-9-CM codes 456.0 or 456.0), those examined with panendoscopy, and those who received intravenous PPIs during hospitalization. In addition, we excluded patients taking more than the standard doses of oral PPIs, because high-dose oral PPIs are always used for active or recent gastrointestinal bleeding. As in previous studies, the standard dose of oral PPIs in our study was defined as omeprazole 20 mg, rabeprazole 20 mg, lansoprazole 30 mg, pantoprazole 40 mg, or esomeprazole 40 mg [17]. This is also the common dosage of PPIs in clinical practice in Taiwan. In Taiwan, the cost of PPIs has been covered by the Taiwan National Health Insurance program on gastric acid-related diseases, such as peptic ulcer disease or reflux esophagitis. The report of panendoscopy should be provided for medical payment for at least four months of PPI therapy. Because a new PPI prescription during hospitalization was excluded initially for possible UGIB, it is reasonable to consider that all of the enrolled cases received PPIs therapy before hospitalization.

Among the remaining patients, those taking oral PPIs, including esomeprazole, lansoprazole, omeprazole, pantoprazole, and rabeprazole, were selected as the study group (PPI group). For this study, death was defined as withdrawal of the patient from the NHI program. During the study period, more than 98% of the Taiwan population was covered by the National Health Insurance program. We also double-checked the defined death cases in the dataset. The patients in this study were followed for 90 days from admission, a period based on the four months of medical payment for PPI therapy in Taiwan.

### Statistical analyses

We used SPSS statistical package (IBM SPSS System for Windows, version 22.0, IBM Corp., Armonk, NY) to perform all analyses. After selecting the pneumonia population in the dataset, we identified the PPI group and developed a multivariate logistic regression model by using stepwise negative selection to generate propensity scores for the PPI group versus a control group (the non-PPI group). Some covariates of the patients in the PPI-group and non-PPI group were heterogeneous, which may have affected the overall mortality rates. To decrease the difference between groups, we performed one-to-three matching by means of the nearest neighborhood matching and caliper matching, with a caliper width set as 0.2. We used this one-to-three propensity score matching to select a control group (non-PPI group) according to age, gender, alcoholism (ICD-9-CM codes 291, 303, 305.00–305.03, and 571.0–571.3), hepatocellular carcinoma (ICD-9-CM code 155.0), hepatic encephalopathy (ICD-9-CM code 572.2), ascites (ICD-9-CM code 789.5 or procedure code 54.91), and renal function impairment (ICD-9-CM codes 584, 585, 586, and 572.4, or other procedure codes relate to renal failure). Comorbid disorders were considered if the condition was found upon discharge.

The Chi square test or Fisher’s exact test was used to compare categorical variables. The Student’s t-test was used to compare continuous variables. The proportional hazards Cox regression model was used to control for the confounding effect of other comorbid factors. We also reported the hazard ratio (HR) and 95% confidence interval (CI) using a significance level of 0.05. We used Kaplan-Meier curve to evaluate the cumulative survival of cirrhotic patients with respiratory infections in the PPI and non-PPI groups. To evaluate later mortality, we calculated the 30 to 90-day day mortality of the patients surviving more than 30 days. In subgroup analysis, the Kaplan-Meier curve was also used for cirrhotic patients with respiratory infections who used different oral PPIs. To evaluate the effect on mortality, patients taking each kind of oral PPI were compared to the non-PPI group. To evaluate later mortality, we also calculated the 30 to 90-day day mortality of patients surviving more than 30 days by kind of PPI, compared to the non-PPI group.

## Results

**Fig 1:**
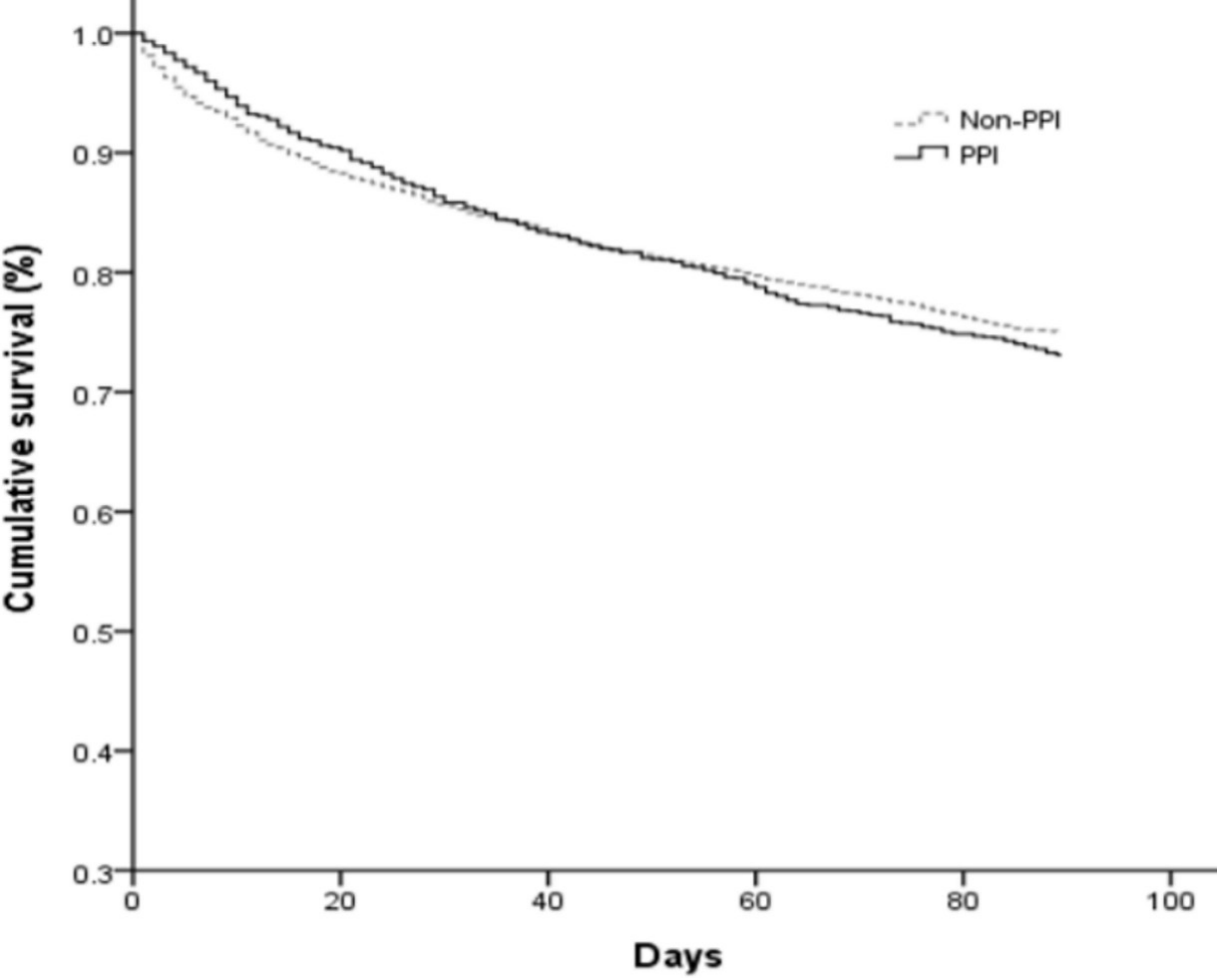
Cumulative survival plot for cirrhotic patients with pneumonia and between the PPI and Non-PPI groups.

The dataset contained 32,898 cases of cirrhotic patients with pneumonia. After excluding patients with active peptic ulcer bleeding or esophageal variceal bleeding; and those receiving panendoscopy, intravenous PPIs, or oral high-dose PPIs, we enrolled a total of 6,432 cirrhotic patients with pneumonia and without active gastrointestinal bleeding in this study. Of these patients, 1,201 cirrhotic patients with pneumonia were also receiving oral PPIs (PPI group). After 1:3 propensity score matching, we enrolled 3,603 cirrhotic patients with pneumonia but not receiving PPIs as the non-PPI group. Table 1 shows the demographic characteristics of the PPI and non-PPI groups. The overall 30-day and 90-day all-cause mortality rates were 13.7% and 26.9% in the PPI group, and 14.3% and 25.1% in the non-PPI group, respectively. After the Cox regression model was adjusted for age, gender, and other comorbid disorders, the HR of oral PPIs for 30-day mortality of cirrhotic patients with pneumonia and without active gastrointestinal bleeding was 0.94 (95% CI, 0.79–1.12, *P* = 0.468) compared to the non-PPI group. The adjusted HR of oral PPIs for 90-day mortality was 1.07 (95% CI, 0.95–1.22, *P* = 0.274) compared to the non-PPI group. The cumulative survival plot is shown in Fig 1. Other statistically significant prognostic factors are shown in Table 2.

**Table 1:** Demographic characteristics of cirrhotic patients with pneumonia, with and without concomitant use of PPIs.

	PPI group (n = 1201)	Non-PPI group (n = 3603)	*P-*value
Male, n (%)	852 (70.9)	2586 (71.8)	0.580
Age (yr)	62.97 ± 14.68	63.01 ± 15.17	0.927
HCC, n (%)	257 (21.4)	829 (23.0)	0.248
Ascites, n (%)	220 (18.3)	633 (17.6)	0.556
RFI, n (%)	84 (7.0)	216 (6.0)	0.215
Hepatic encephalopathy, n (%)	184 (15.3)	493 (13.7)	0.158
Alcoholism, n (%)	340 (28.3)	1019 (28.3)	0.985

Abbreviations: PPI, proton pump inhibitor; HCC, hepatocellular carcinoma; RFI, renal function impairment.

**Table 2:** Adjusted hazard ratios of the risk for 30-day mortality of cirrhotic patients with pneumonia without active gastrointestinal bleeding.

Variable	Hazard ratio	95% confidence interval	*P-*value
Age	1.03	1.02–1.03	<0.001
Male	1.27	1.07–1.52	0.007
Alcoholism	0.94	0.75–1.17	0.564
Ascites	1.52	1.28–1.81	<0.001
Hepatic encephalopathy	1.81	1.50–2.18	<0.001
Hepatocellular carcinoma	1.93	1.65–2.27	<0.001
RFI	2.01	1.59–2.56	<0.001
Oral PPI use	0.94	0.79–1.12	0.468

Abbreviations: RFI, renal function impairment; PPI, proton pump inhibitor.

To evaluate later mortality, we calculated the 30 to 90-day mortality rate of patients surviving more than 30 days. The 30 to 90-day mortality rate was 15.3%, in the PPI group and 12.6% in the non-PPI group. After adjusting the Cox-regression model for age, gender, and other comorbid disorders, the HR of oral PPIs on 30 to 90-day mortality was 1.26 (95% CI, 1.05–1.52; P = 0.013).

**Fig 2:**
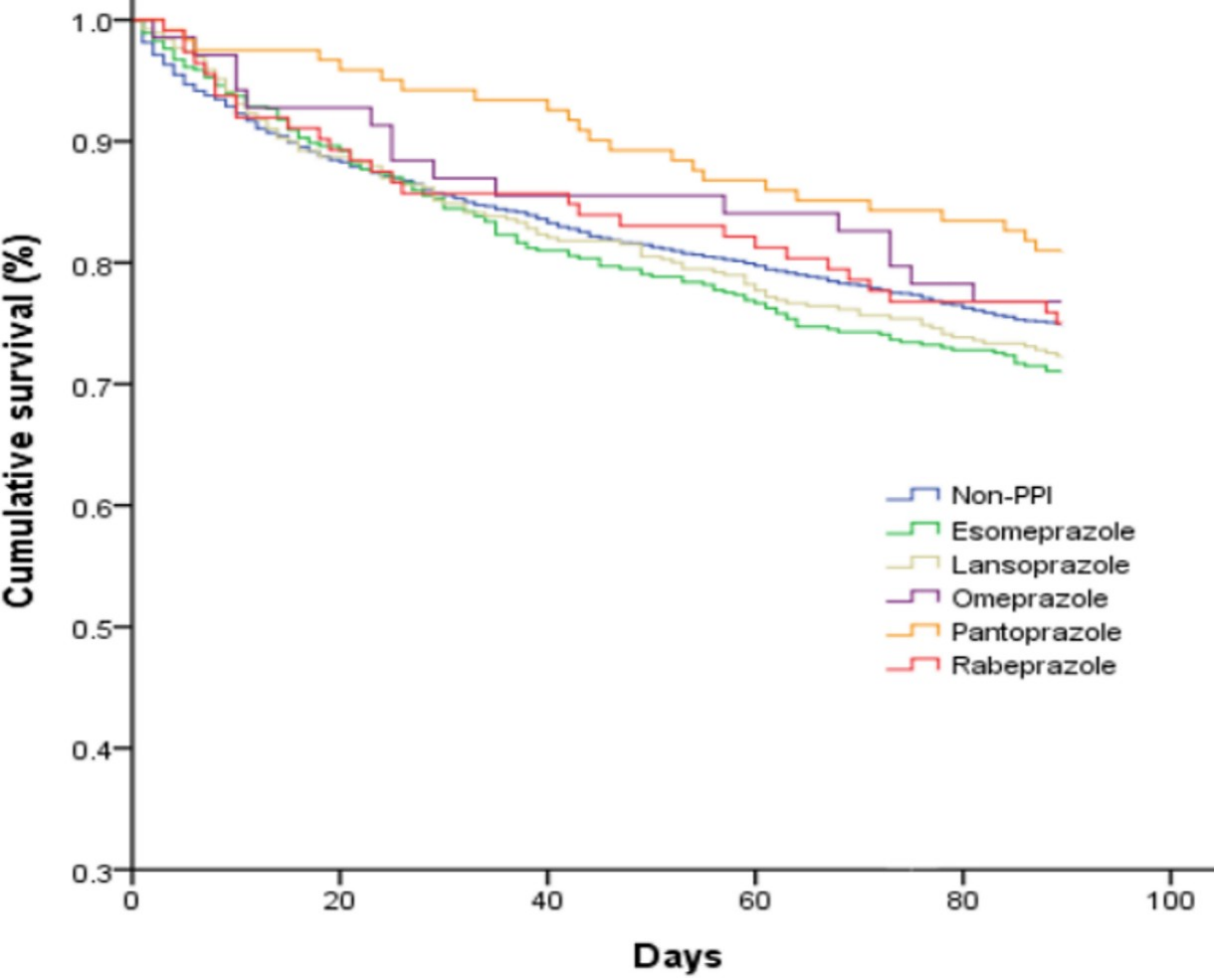
Cumulative survival plot for cirrhotic patients with pneumonia who were taking different oral proton pump inhibitors.

To evaluate the effect of each kind of oral PPI on the mortality of cirrhotic patients with pneumonia but without active gastrointestinal bleeding, the mortality rate associated with each kind of oral PPI was compared to that of the non-PPI group. The patients receiving only one kind of oral PPI during hospitalization were enrolled (Table 3). Oral esomeprazole was associated with a significantly increased 30 to 90-day mortality risk (HR, 1.33; 95% Cl, 1.03–1.73; *P* = 0.031). None of the other oral PPIs had any effect on 30 to 90-day mortality. The cumulative survival plot by individual PPI is shown in Fig 2.

**Table 3:** Adjusted HRs of different PPIs for 30-day and 30 to 90-day mortality of cirrhotic patients with pneumonia and no active gastrointestinal bleeding.

	30-day mortality		30- to 90-day mortality	
	Case/control	HR (95% CI)	Case/Control	HR (95% CI)
PPIs	1201/3603	0.94 (0.79–1.12)	1037/3088	1.26 (1.05–1.52)
Esomeprazole	463/3603	1.00 (0.78–1.29)	395/3088	1.33 (1.03–1.73)
Lansoprazole	390/3603	1.01 (0.77–1.33)	332/3088	1.23 (0.91–1.65)
Omeprazole	69/3603	0.93 (0.48–1.80)	60/3088	1.09 (0.52–2.32)
Pantoprazole	121/3603	0.36 (0.14–0.77)	114/3088	1.12 (0.68–1.84)
Rabeprazole	112/3603	0.93 (0.57–1.54)	96/3088	1.09 (0.61–1.94)

Abbreviations: HR, hazard ratio; PPI, proton pump inhibitor; CI, confidence interval.

## Discussion

This nationwide population-based study was designed to identify the association of PPIs with mortality among patients with cirrhosis and pneumonia, but no active gastrointestinal bleeding. Using the NHIRD, we enrolled a large population to provide reliable information that could be applied to clinical practice. Our study showed that PPIs were not associated with an increased risk of 30-day mortality among cirrhotic patients with pneumonia who did not have active gastrointestinal bleeding. However, the prolonged use of PPIs may correlate with a greater risk of mortality than that of non-PPI users.

Currently, omeprazole, esomeprazole, lansoprazole, pantoprazole, and rabeprazole are available in Taiwan. PPIs are used mainly to treat gastric acid-related diseases, including peptic ulcer disease, erosive esophagitis, and gastroesophageal reflux disease. PPIs are also frequently used in cirrhotic patients with acid-related diseases [7, 8]. However, some studies have confirmed the potential risk of increased incidence of some serious infectious diseases like clostridium difficile, spontaneous bacterial peritonitis, or respiratory infections [18–20]. PPIs can markedly reduce gastric acid secretion, which is important in inhibiting bacterial overgrowth in the gastrointestinal tract. Without the effect of gastric acid, infectious diseases such as pneumonia are more likely. Several studies have also reported a higher risk of pneumonia after the initiation of PPIs therapy [11, 12].

Pneumonia is life-threatening in patients with cirrhosis. Because of the frequent use of PPIs in cirrhotic patients and the potentially increased risk of developing respiratory infections, it is worthwhile to know the association of PPIs with mortality in cirrhotic patients with pneumonia. In our study, PPIs were not associated with an increase in 30-day mortality in cirrhotic patients with pneumonia. In clinical practice, the use of antibiotics is inevitable in cirrhotic patients with bacterial pneumonia. According to the type of antibiotic used, the disadvantage of reducing gastric acid secretions by PPIs may not be a problem. The use of antibiotics during hospitalization, then, may be the reason that PPIs do not have a significant association with 30-day mortality. However, the 30 to 90-day mortality is a different story. In this study, PPIs may correlate with higher 30 to 90-day mortality rates. During this period, patients are usually discharged and may not need antibiotics for infection control. However, prolonged PPI therapy may correlate with higher mortality due to bacterial overgrowth in the gastric or intestinal tract related to strong gastric acid suppression. However, further study is still needed on this topic. At least from our study, we believe PPI therapy may be safe for cirrhotic patients with pneumonia. However, without the coverage of antibiotics, PPIs therapy may be correlated with higher mortality in these patients.

Our study results also showed that esomeprazole causes higher 30 to 90-day mortality among cirrhotic patients with pneumonia without active gastrointestinal bleeding than do other PPIs, compared to those not taking PPIs. According to the pharmacokinetic and pharmacodynamics characteristics of PPIs, the use of esomeprazole maintains the longest duration of pH levels >4, and produces the highest mean pH of the PPIs [17, 21]. In other words, esomeprazole has the highest potential for increasing gastric pH. This characteristic may contribute to a higher risk of SIBO and increased risk of cirrhosis-related complications. This may be the reason why esomeprazole was associated with a higher risk for long-term mortality among cirrhotic patients. However, more evidence will be needed to prove this theory.

There were several limitations in our study. First, our study did not measure the severity of cirrhosis by using the Mayo Clinic model for end-stage liver disease score or the Child-Pugh score. This dataset does not provide either the detail of renal function or quantify hepatic encephalopathy. The dataset also did not provide biopsy-proven data or other confirmed images to confirm liver cirrhosis. This was an essential disadvantage, because the dataset provided no laboratory data, such as bilirubin levels, albumin levels, or prothrombin times. Second, we could not identify the exact etiology of non-alcoholic cirrhosis through the dataset. This is because the cause of cirrhosis is not always coded in patient hospitalizations. Also, alcohol consumption is not quantified in alcoholic cirrhotic patients. However, previous studies have established that the etiology of non-alcohol-related cirrhosis in Taiwan is primarily related to infection with the hepatitis B virus [22]. Third, we did not know the duration of a patient’s exposure to PPIs before admission. However, PPI use 48 hours after the initial dose has been demonstrated to induce increased and sustained gastric acid suppression [17]. Fourth, there was no information about the etiology and severity of respiratory infections. Also, the cause of death could not be provided in the dataset we used. Finally, we could not know how long the patient received PPIs after discharge. Although PPIs are mainly used for gastric acid-related diseases, the dataset could not provide the exact reason for using PPIs. Therefore, the effect of the duration of oral PPI use on the mortality of cirrhotic patients is still unknown.

In conclusion, this nationwide population-based study showed that concomitant oral PPI use was not associated with 30-day mortality among cirrhotic patients with pneumonia who did not have active gastrointestinal bleeding. However, prolonged PPI therapy may be associated with higher mortality rates than in non-PPI users.
